# Traditional Chinese Medicine JingYinGuBiao Formula Therapy Improves the Negative Conversion Rate of SARS-CoV2 in Patients with Mild COVID-19

**DOI:** 10.7150/ijbs.76699

**Published:** 2022-09-01

**Authors:** Bowu Chen, Peihua Geng, Jiaojiao Shen, Suthat Liangpunsakul, Hua Lyu, Jue Zhang, Yanbing Yang, Lei Zhang, Yuping Xu, Chunling Dong, Yanping Wang, Yan Xue, Wei Zhang, Hua Liu, Man Li, Yueqiu Gao

**Affiliations:** 1 Department of Hepatopathy, Shuguang Hospital, Affiliated to Shanghai University of Traditional Chinese Medicine, Shanghai, China.; 2 Medical Department, Shuguang Hospital, Affiliated to Shanghai University of Traditional Chinese Medicine, Shanghai, China.; 3 Nursing Department, Shuguang Hospital, Affiliated to Shanghai University of Traditional Chinese Medicine, Shanghai, China.; 4 Division of Gastroenterology and Hepatology, Department of Medicine, Indiana University, USA.; 5 National Monitoring Center for Medical Services Quality of TCM Hospital, Shanghai, China.; 6 Clinical Laboratory, Shuguang Hospital, Affiliated to Shanghai University of Traditional Chinese Medicine, Shanghai, China.; 7 General Affairs Department, Shuguang Hospital, Affiliated to Shanghai University of Traditional Chinese Medicine, Shanghai, China.; 8 Respiratory Department, Shuguang Hospital, Affiliated to Shanghai University of Traditional Chinese Medicine, Shanghai, China.; 9 Division of Service supervision of Traditional Chinese Medicine, Shanghai Municipal Health Commission, Shanghai, China.; 10 Laboratory of Cellular Immunity, Institute of Clinical Immunology, Shuguang Hospital, Affiliated to Shanghai University of Traditional Chinese Medicine, Shanghai, China.; 11 Institute of Infectious diseases of integrated traditional Chinese and Western medicine, Shanghai, China.

**Keywords:** COVID-19, traditional Chinese medicine, JingYinGuBiao formula, negative conversion rate, negative conversion time

## Abstract

**Background:** Traditional Chinese Medicine (TCM) JingYinGuBiao formula (JYGB) was recommended by the Expert consensus on Traditional Chinese Medicine diagnosis and treatment of COVID-19 infection in Shanghai. We evaluated the safety and efficacy of JYGB in treating mild COVID-19 patients.

Methods: A prospective, double-blind, randomized, controlled trial was conducted (ClinicalTrial.gov registration number: ChiCTR2200058695). A total of 885 patients were randomized into the treatment group (administration of JYGB,n=508) or the control group (administration of TCM placebo, n=377) with 7-day treatment. The primary outcomes were the negative conversion rate and negative conversion time of SARS-CoV2 RNA. Secondary outcomes included the hospitalized days and symptom improvement.

**Results:** A total of 490 and 368 patients in the treatment and control groups completed the study. The cumulative negative conversion rates at 2 days, 3 days, 4 days, and 6 days post randomization in the treatment group were all markedly higher than those in the control group (13.88% vs. 9.24%, *P*=0.04; 32.24% vs. 16.58%, *P*<0.001; 51.43% vs. 36.14%, *P* <0.001; 77.76% vs. 69.84%, *P*=0.008). Compared with the control group, after JYGB treatment, the median negative conversion time (4.0 [3.0-6.0] vs. 5.0 [4.0-7.0] days, *P*<0.001) and hospitalized days (6.0 [4.0-8.0] vs. 7.0 [5.0-9.0] days, *P*<0.001) were reduced. While the symptoms were improved, there were no significant differences in symptom disappearance rates between both groups. In addition, further sub-group analysis showed that for patients with interval time ≤4 days or patients≤ 60 years, the clinical effects of JYGB were more remarkable with an increase in cumulative negative conversion rates, a decrease in negative conversion time and hospitalized days. JYGB was well tolerated without any severe side effects.

**Conclusion:** JYGB, a TCM prescription, improves the negative conversion rate of SARS-CoV2 in mild COVID-19 patients.

## Introduction

Coronavirus disease 2019 (COVID-19) is a new pandemic that was declared by the World Health Organization (WHO) on March 11, 2020 1. COVID-19 is caused by severe acute respiratory syndrome coronavirus 2 (SARS-CoV-2) and has quickly spread worldwide since December 2019. As of May 2022, there have been 520,372,492 confirmed cases of COVID-19, including 6,270,232 deaths, reported to the WHO. This number likely has been underestimated because asymptomatic viral carriers and patients with mild diseases with linsidious or atypical symptoms and signs have not been tested or reported. Despite the reduction of SARS-Cov-2 infection after vaccination or infection, the COVID-19 pandemic continues to affect people worldwide.

In the early 2022, the highly transmissible Omicron variant has rapidly replaced other circulating variants in almost all countries. Due to its distinctive features in etiology, epidemiology, and pathology, this infectious disease poses considerable diagnostic and therapeutic challenge. Paxlovid is the only authorized drug for emergency use by the Food and Drug Administration to treat mild to moderate COVID-19 in people aged 12 and older who are at a high risk of serious illness 2,3,4. However, there are no authorized medicines for patients with mild COVID-19.

Traditional Chinese medicine (TCM) has been used to combat more than 500 outbreaks of pestilence in a long history since the first pandemic in 243 BC recorded in Shi Ji (Historical Records) to 1949. COVID-19 falls under the category of "pestilence". In light of the long history of evolution and the proven efficacy in patients with influenza 5,6, TCM has recently been repurposed for the clinical management of COVID-19 7,8. There are promising data on the benefits of TCM in reducing the disease exacerbation rate for mild and moderate cases of COVID-19 in the WHO Expert Meeting on Evaluation of Traditional Chinese Medicine in the Treatment of COVID-19. TCM scheme has been included in the guideline on diagnosis and treatment of COVID-19 (Trial 9th edition) released by National Health Commission of the People's Republic of China 9.

TCM-JingYinGuBiao formula (JYGB) originated from two classical prescriptions including YinQiaoSan and YuPingFeng powder. The YinQiaoSan, as a famous prescription of Wu Jutong, a doctor in the Qing dynasty, is mainly used for the treatment of influenza, hand-foot-mouth disease, esophagitis, pneumonia, acute tonsillitis, mumps, and other viral infections 10,11. YuPingFeng powder originates from the book "Danxi′s Experiential Therapy" written by Zhu Danxi (1281 A.D.-1358 A.D.), and is used for the cure and prevention of diseases related to immunodeficiency, such as relapses of respiratory infection, allergic rhinitis, and chronic bronchitis 12. From February 26, 2022 to April 23, 2022, the cumulative number of locally confirmed COVID-19 infections in Shanghai was 488,607. Patients with asymptomatic infections and mild COVID-19 were enrolled in the mobile cabin hospitals. The pathological nature in TCM of COVID-19 mainly shows dampness, heat, and toxin. Most of the patients infected with Omicron variant strain are characterized by wind-heat attacking lung and deficiency of Lung Qi. JYGB is mainly used to dispel wind, clear heat, and detoxify, and also benefit Qi and solidify the surface. JYGB, one of the hospital preparations of Shuguang hospital (No.SYZ-QB-0004-2022), has been recommended to treat patients with mild and moderate COVID-19 by the Expert consensus on TCM diagnosis and treatment of Coronavirus infection in Shanghai (2022 Spring Edition). However, no existing studies with a sufficient sample size and prospective randomized designs have been conducted to evaluate the safety and efficacy of JYGB for the treatment of COVID-19.

In the current study, we conducted a prospective, double-blind, randomized, placebo-controlled trial to explore the safety and efficacy of JYGB in treating patients with mild COVID-19. The objectives of the study were to determine if the administration of JYGB resulted in an increase in the negative conversion rate, shortened negative conversion time of SARS-CoV2 RNA, and improved COVOD-19 symptoms.

## Methods

### Study design

We conducted a prospective, double-blind, randomized, placebo-controlled trial during COVID-19 Omicron epidemic from April 8, 2022 to May 6, 2022 in Shanghai in China (ClinicalTrial.gov registration number: ChiCTR2200058695). The protocol and consent forms were reviewed and approved by the IRB of Shuguang Hospital affiliated with Shanghai University of TCM (No. 2022-1095-32-01). The study was performed in accordance with the principles of Declaration of Helsink and all participants signed written informed consent forms before enrollment.

### Patient enrollment

Patients with mild COVID-19 were enrolled. All patients were admitted to a mobile cabin hospital, where they were quarantined and observed. Patients who fulfilled all of the following criteria were included: (1) the diagnostic criteria for COVID-19; (2) 18 to 80 years old; (3) mild disease as defined by mild symptoms without any evidence of pneumonia on radiographic imaging. The diagnosis and classification of COVID-19 were defined according to Guidelines for the Diagnosis and Treatment of Coronavirus Disease 2019 (Trial 9th edition) 9.

Patients were excluded if they fulfilled one of exclusion criteria: (1) patients who could not receive the treatment of TCM; (2) patients who are sensitive to or intolerant of the composition of TCM; (2) patients with uncontrolled severe cardiovascular and metabolic disease; (3) patients with a mental or severe psychiatric disorder; (4) women of child-bearing age with positive pregnancy test results or those in the lactating period; (5) patients whose condition was further complicated with other active infections.

### Drug administration and randomization

The TCM formula that was used in our study was JYGB, which is composed of 10 herbs: 9g jinyinhua (Lonicera japonica Thunb), 9g jingjie (Herba Schizonepetae), 12g huangqi (Astragalus propinquus Schischkin), 9g fangfeng (Saposhnikovia divaricate), 9g huoxiang (Agastache rugosus), 9g banlangen (Isatis Root), 6g jiegeng (Platycodon Grandiflorum), 15g lugen (rhizoma phragmitis), 9g baishu (Atractylodes macrocephala Koidz), and 9g gancao (GlycyrrhizauralensisFisch). The TCM placebo was used as control in this study, which contained 1g huoxiang and 1g gancao in order to have a similar color and taste (brown and bitter) with JYGB 13. Table S1 lists the names of these herbs in Chinese script and English translation.

The criteria for the quality of the herbs we used were in accordance with the 2020 Chinese pharmacopoeia 14. The concentrated granules of JYGB and TCM placebo were prepared and provided by Shanghai Wanshicheng State Medicine Products Co., Ltd. Herbs were extracted successively twice with boiled water. The extract was then filtered through absorbent gauze, and the filtrate was concentrated to plaster and then dried to produce the extract granules. Before the study, the granules were tested for heavy metals, microbial contamination, and residual pesticides. In addition, samples were tested by the thin-layer identification method. Both TCM granules and the control samples can show the spots of the same color at RF value. The quality control data of herbs was shown in Figure S1. All results met quality and safety standards in China.

An ultra-high performance liquid chromatography-quadrupole/Orbitrap high resolution mass spectrometry (UHPLC-Q-Orbitrap HRMS) was used for the identification analysis of the components in JYGB granules, and the multistage fragments ions data was compared with the standard substance and literature consulting. The main components in JYGB granules included Astragaloside IV, Calycosin-7-glucoside, Prim-O-glucosylcimifugin, 4'-O-beta-Glucopyranosyl-5-O-Methylvisamminol, Pulegone, 4-Coumaric acid, Platycodin D, Cynaroside, Chlorogenic acid, 3,5-O dicaffeoylquinic acid, 4,5-Dicaffeoylquinic acid, Liquiritin, Glycyrrhizic acid, Acacetin, (R, S)-Goitrin, fructose, and Sucrose, which were shown in Table S2 and Figure S2. Laboratory workers were blinded to the identity of the granules.

After agreeing to participate, signing the informed consent form, and completing the baseline visit, all patients were randomly assigned to either the control or treatment group by using a cluster randomization. A statistician who was not involved in data collection nor analysis produced the randomization list. Participants in the treatment group were administrated JYGB granules orally for 15g twice daily for 7 days, and participants in the control group were administrated TCM placebo orally for 15g twice daily for 7 days. An investigator who was blinded to the participants' characteristics assigned the participants to each treatment group.

All participants were hospitalized so that they could be quarantined and closely observed and were followed until discharge. The criteria for discharge were when patients had 2 consecutive negative RT-PCR results separated by 24 hours apart, according to the Guidelines for the Diagnosis and Treatment of Coronavirus Disease 2019 (Trial 9th edition) 9. Adherence to therapy was assessed by nurses who were blinded to the study.

### Assessment

During hospitalization, nurses who were blinded to the study used a forehead thermometer to measure participants' body temperature daily at 6 a.m. and 6 p.m. The presence of COVID-19 symptoms (cough, hypodynamia, headache, stuffy nose, runny nose, pharyngalgia, myalgia, chest distress, vomition, abdominal distension, stomachache, and diarrhoea) and drug-associated side effects were recorded before and after treatment. The baseline data including age, stature, body weight, and comorbidity were collected using an electronic questionnaire administered over the internet.

The primary objective of the trial was to assess the efficacy of JYGB as compared with control by comparing the negative conversion rate and negative conversion time of SARS-CoV-2 RNA. Other clinical effectiveness included the hospitalized days and the disappearance rate of COVID-19 symptoms. Safety outcomes included adverse events and serious adverse events that occurred during the study. The negative conversion time of SARS-CoV-2 RNA was defined as the time from randomization to the first day of at least 2 consecutive negative RT-PCR results separated by 24 hours apart. SARS-CoV-2 was considered negative if the Ct values of the ORF1ab gene and the N gene were equal or over 35 9.

### Statistical analysis

All data were analyzed by SPSS version 25.0 for Windows and MedCalc software (version 20.027). Data were presented as number and percentages for categorical variables. Continuous variables with non-normal variables were reported as the median and interquartile range (M [Q1-Q3]). Two independent sample t-test, Mann Whitney rank sum test, and chi-square test were used. Kaplan-Meier method was used to analyze the time to the primary endpoint with a log-rank test. Cox proportional-hazards model was used to assess the risk factors on the negative conversion of SARS-CoV2. All tests were two-tailed, and *P* ≤ 0.05 was considered statistically significant.

## Results

### Participant characteristics

**Figure 1:**
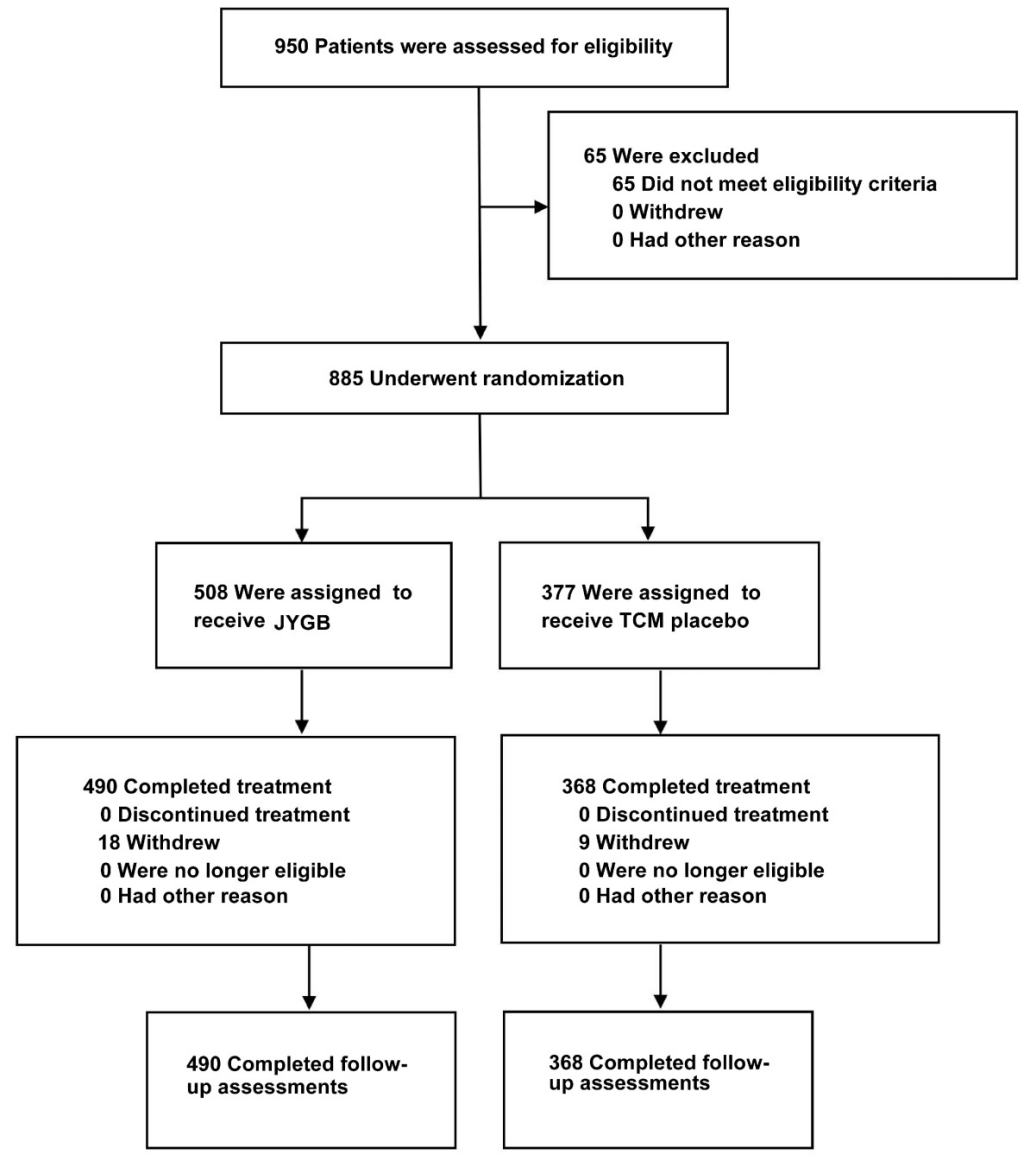
** Study flow diagram.** A total of 950 patients were enrolled. Sixty-five patients were excluded from the study. Eight hundred and eighty-five patients were randomly assigned to receive JYGB (508, Treatment group) or TCM placebo (377, control group). After the randomization, 18 patients were excluded from the treatment group and 9 patients were excluded from the control group because they refused to provide the relevant data. A total of 490 patients in the treatment group and 368 patients in the control group completed the study.

A total of 950 patients were enrolled between April 8 and May 6, 2022 in mobile cabin hospital of City footprint hall in Shanghai. Of these, 65 patients were excluded because they did not meet the inclusion criteria. Eight hundred and eighty-five patients were randomly assigned to receive JYGB (n=508, treatment group) or TCM placebo (n=377, control group). After the randomization, 18 patients were excluded from the treatment group and 9 patients were excluded from the control group because they refused to provide the relevant data. A total of 490 patients in the treatment group and 368 patients in the control group completed the study. The disposition of the study participants was shown in Figure 1.

The baseline characteristics of the study cohort are outlined in Table 1. At baseline, there were no differences in median age (48.0 vs. 49.0 years old) and body mass index (23.32 vs. 23.63 kg/m^2^) between control and treatment groups. The proportion of patients with COVID-19 related symptoms was comparable between both groups except for the proportion of patients reporting headache (14.67% vs. 10.00%, *P*=0.04) and pharyngalgia (31.52% vs. 24.28%, *P*=0.02). Patients randomized into the treatment group had higher prevalence of concurrent medical comorbidities (15.31% vs. 7.07%, *P*<0.001). The median interval time between the onset of illness and the time of randomization was 3.0 [2.0, 4.0] days in the control group and 3.0 [2.0, 4.0] in the treatment group (*P*=0.01). The average interval time between the onset of illness and the time of randomization was 2.83±1.65 days in the control group and 3.30±1.87 days in the treatment group (*P*<0.001). There was no difference in the baseline Ct value for the *ORF* genes in patients randomized to the treatment group when compared to that in the control group (25.67[22.15, 30.10] vs. 25.26[21.32 vs. 29.76], *P*=0.29). However, the Ct value for *N* gene was higher in patients assigned to the treatment group (25.40[21.74, 29.89] vs. 24.49[20.28, 28.81], *P*=0.004).

### Clinical outcomes

**Figure 2:**
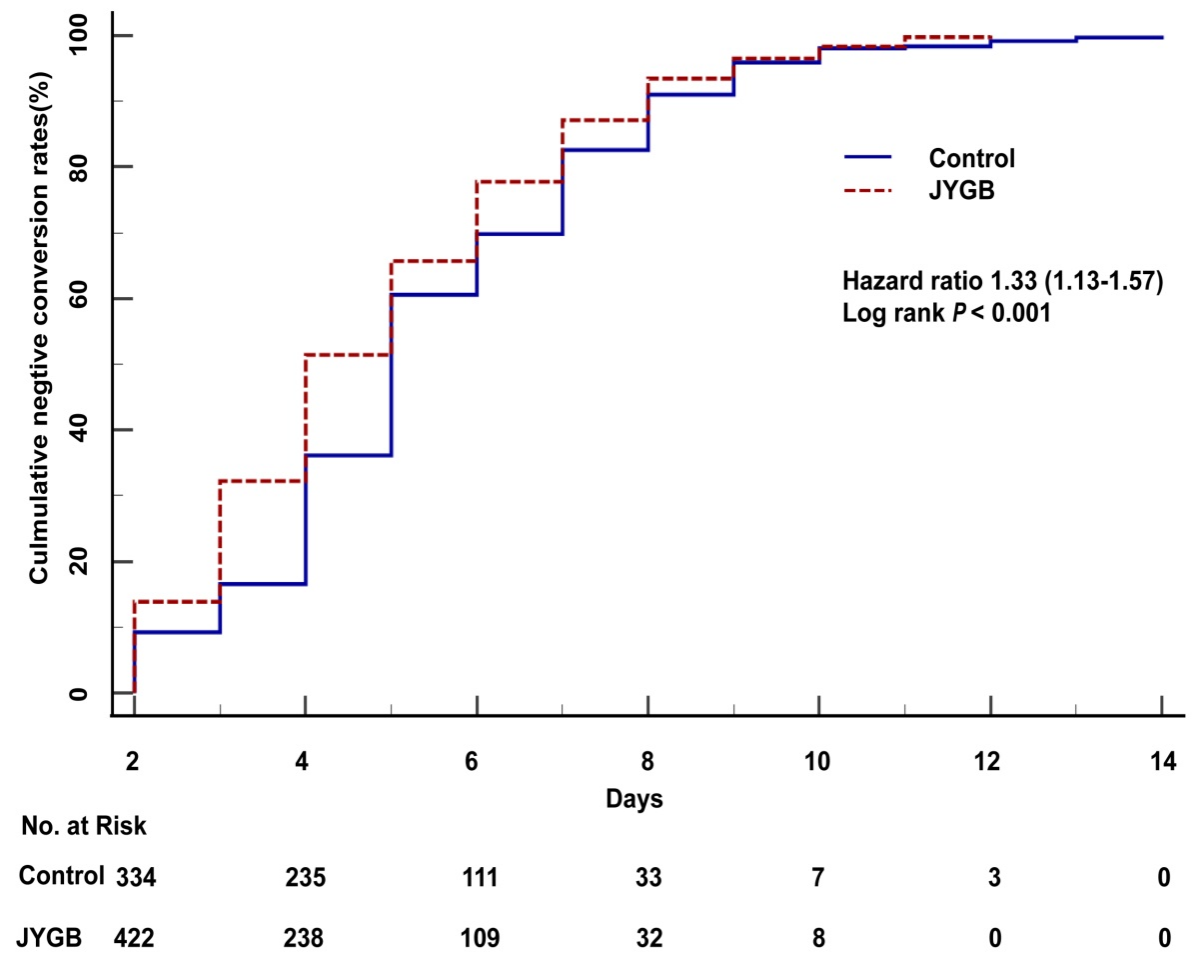
**The overall survival analysis in all patients.** The overall survival analysis was executed in the treatment group (JYGB) and the control group (Control). Kaplan-Meier method was used to analyze the time to the primary endpoint with a log-rank test.

The cumulative negative conversion rates at 2 days, 3 days, 4 days, and 6 days post randomization in the treatment group were all higher than those in the control group (13.88% vs. 9.24%, *P*=0.04; 32.24% vs. 16.58%, *P*<0.001; 51.43% vs. 36.14%, *P* <0.001; 77.76% vs. 69.84%, *P*=0.008), as shown in Table 2. In the log-rank analysis, patients who were randomized to the treatment group had approximately 1.33 fold [95% CI 1.13-1.57, *P*<0.001] higher than that in the control group to achieve a negative conversion (Figure 2). A significant reduction in the median negative conversion time was seen in the treatment group compared with the control group (4.0 [3.0,6.0] vs 5.0 [4.0,7.0] days, *P*<0.001), as shown in Table 3. The median hospitalized days in the treatment group were significantly shorter than those in the control group (6.0 [4.0-8.0] vs 7.0 [5.0-9.0] days, *P*<0.001), as shown in Table 3.

We observed an improvement in clinical symptoms and symptom disappearance rates were analyzed in both groups during the study period, including fever (96.23% vs. 94.29%), cough (77.82% vs. 70.97%), hypodynamia (91.46% vs. 85.51%), headache (93.88% vs. 96.30%), stuffy nose (86.51% vs. 94.05%), runny nose (93.62% vs. 94.67%), pharyngalgia (88.24% vs. 90.52%), myalgia (96.88% vs. 92.86%), chest distress (92.86% vs. 96.03%), vomition (100.00% vs. 100.00%), abdominal distension (100.00% vs. 100.00%), stomachache (100.0% vs. 100.0%), and diarrhoea (95.45% vs. 83.33%). However, there were no differences in the proportion of patients with the improvement of COVID-19 symptoms among patients who received treatment compared to controls (*P*>0.05), as shown in Supplementary Table S3. None of patients in both groups developed moderate or severe COVID-19 during the study.

### Factors associated with a negative conversion of SARS-CoV2

**Figure 3:**
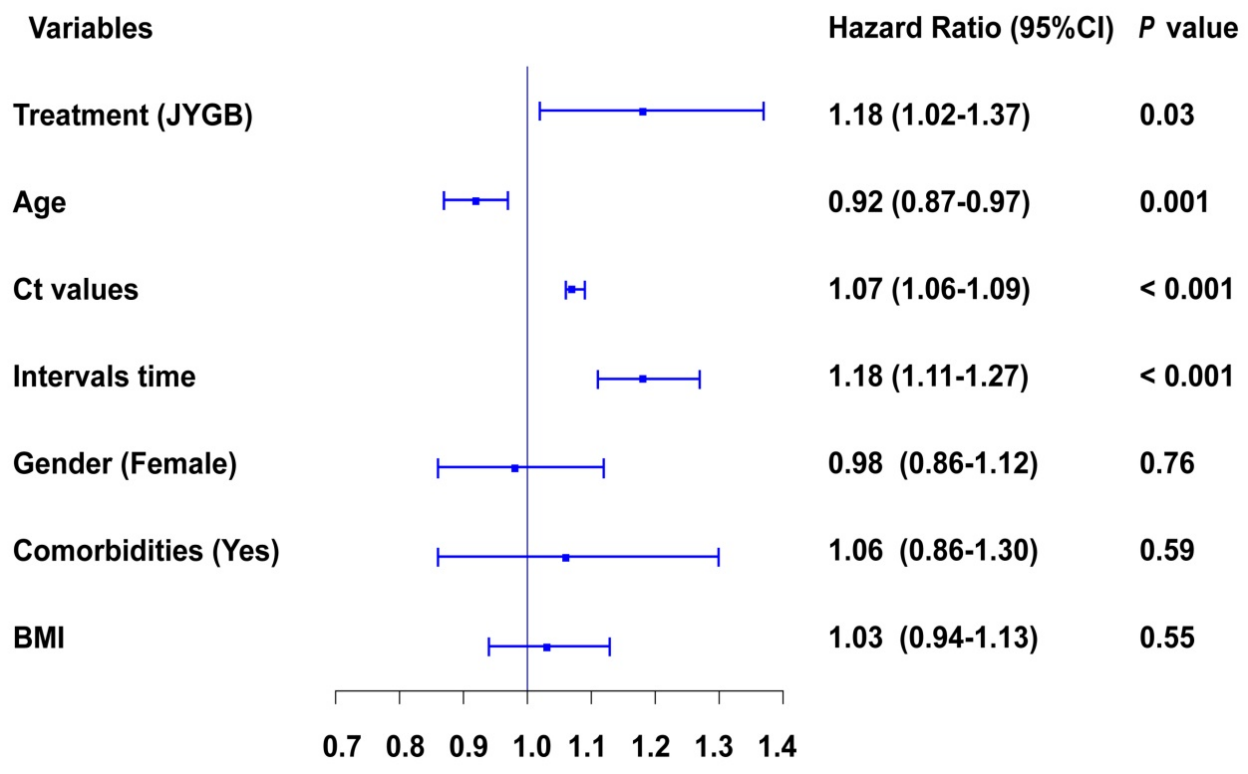
** Cox regression analysis of different variables contribute to the negative conversion of SARS-CoV2 RNA.** The influences of major variables at baseline on the negative conversion of SARS-CoV2 RNA were explored by multivariate Cox regression analyses. &, Ct value of *ORF* gene and Ct value of *N* gene were highly correlated variables, and both of them can't be enrolled into this model at the same time. In this figure, Ct value of ORF was shown, but both of them contributed to the negative conversion of SARS-CoV2.

We next determined variables associated with a negative conversion of SARS-CoV2. The following variables were independently associated with a negative conversion, administration of JYGB [HR 1.18, 95CI% 1.02-1.37, *P*=0.03], age [HR 0.92, 95CI% 0.87-0.97,* P=*0.001], Ct values at baseline [HR 1.07, 95CI% 1.06-1.09, *P*<0.001], and interval time before randomization [HR 1.18, 95CI% 1.11-1.27, *P*<0.001], as shown in Figure 3 and Table S4.

### Age and the negative conversion rate of SARS-CoV2 RNA

According to Guidelines for the Diagnosis and Treatment of Coronavirus Disease 2019(Trial 9th edition), people aged beyond 60 were at high-risk of severe COVID-19 symptoms. When we dichotomized our patients based on the age cut off at 60 years old, we found that for those ≤ 60 years old, the cumulative negative conversion rates at 2 days, 3 days, 4 days, 5 days, and 6 days for those receiving treatment were significantly higher than that in the control group (14.61% vs 9.74%, *P*=0.05; 34.26% vs 17.53%, *P*<0.001; 52.64% vs 38.31%, *P*<0.001; 69.27% vs 62.34%, *P=*0.05; 80.35% vs 72.40%, *P=*0.01), as shown in Table 4. For patients > 60 years old, we only observed the difference in the cumulative negative conversion rates at 4 days (46.24% vs 25.00%, *P=*0.01), as shown in Table 4.

**Figure 4:**
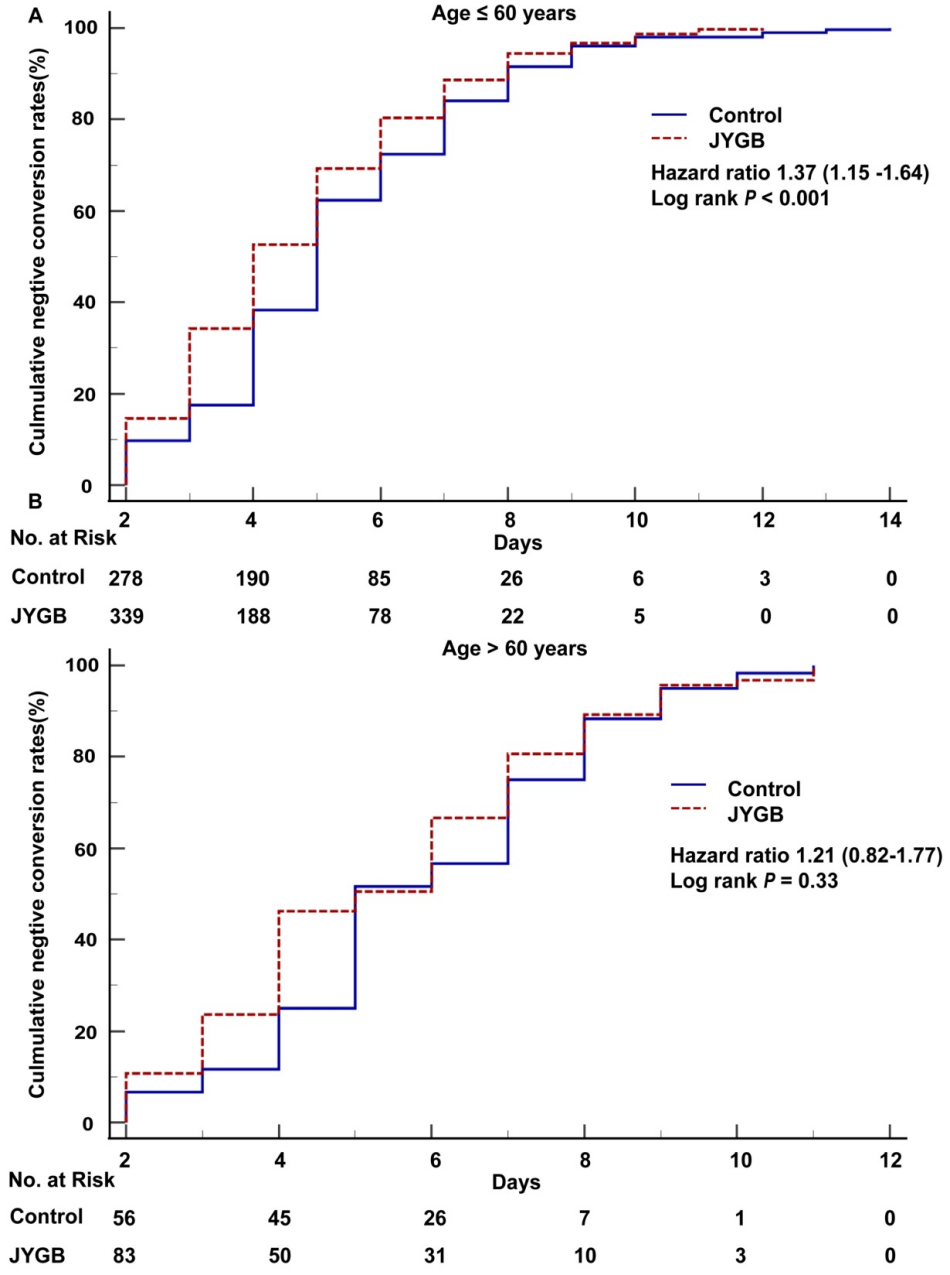
**The overall survival analysis in age subgroups.** Kaplan-Meier method was used to analyze the time to the primary endpoint with a log-rank test. A. The overall survival analysis in age subgroup (age ≤ 60) was executed. B. The overall survival analysis in age subgroup (age > 60) was executed.

In the log-rank analysis for patients ≤ 60 years old, patients who were randomized to the treatment group had approximately 1.37 fold [95% CI 1.15-1.64, *P*<0.001] higher than that in the control group to achieve a negative conversion (Figure 4A). In addition, patients in the treatment group had the shorter median negative conversion time and hospitalized days compared with that in control groups (4.0 [3.0-6.0] vs 5.0 [4.0-7.0], *P*<0.001 and 6.0 [4.0-8.0] vs 7.0 [5.0-9.0], *P*<0.001) (Table 5). We did not observe the difference in the time to negative conversion and the length of hospital stay in those who were > 60 years old (Figure 4B and Table 5).

### Association between interval time from the onset of illness to randomization and the negative conversion rate of SARS-CoV2 RNA

For patients with an interval time from the onset of illness to randomization ≤4 days, the cumulative negative conversion rates at 3 days and 4 days in the treatment group were markedly higher than that in the control group (23.44% vs 13.77%, *P*=0.03; 44.69% vs 31.88%, *P*=0.01, Table 6). For those with the onset > 4 days before randomization, we only observed a significant cumulative negative conversion rate at 3 days (57.45% vs 34.92%, *P*=0.006, Table 6).

**Figure 5:**
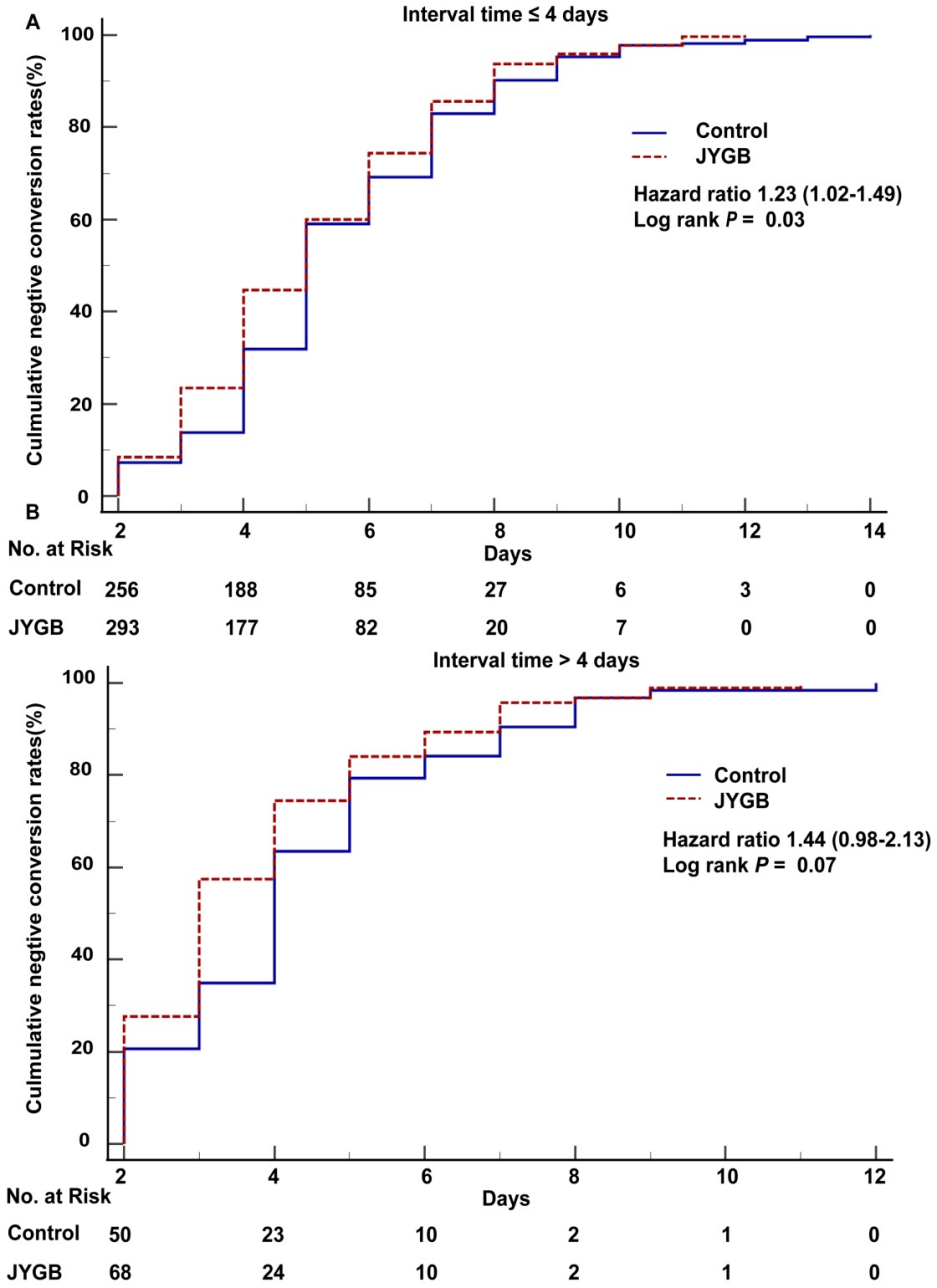
**Negative conversion time and hospitalized days in the subgroups of interval time.** A. The overall survival analysis in subgroup (interval time ≤ 4 days) was executed. B. The overall survival analysis in subgroup (interval time > 4 days) was executed. The interval time is the time between the onset of illness and randomization.

In the log-rank analysis for patients in the subgroup with interval time ≤4 days, patients who were randomized to the treatment group had approximately 1.23 fold [95% CI 1.02-1.49, *P*=0.03] higher than that in the control group to achieve a negative conversion (Figure 5A). As shown in Table 7, the negative conversion time in the treatment group was shorter than that in the control group (median [Q1,Q3] : 5.0 [4.0,7.0] days vs. 5.0 [4.0,7.0] days, *P*=0.02 or mean±SD: 5.16±2.13 days vs. 5.56±2.19 days, *P*=0.03). In addition, the patients in the treatment group had the shorter median hospitalized days (6.0 [5.0,9.0] vs. 7.0 [6.0,9.0], *P=*0.002).

In the subgroup with interval time > 4 days, we did not observe the difference in the number of patients showing negative conversion (HR=1.44, 95% CI [0.98-2.13], *P*=0.07, Figure 5B). In the subgroup with interval time >4 days, the median negative conversion time and the hospitalized days in the treatment group were shortened compared with that in the control group (3.0 [2.0,5.0] vs 4.0 [3.0,5.0], *P*=0.04; 5.0 [4.0,7.0] vs 6.0 [5.0,8.0], *P*=0.02), as shown in Table 7.

### Safety

Three cases in the control group and two cases in the treatment group had diarrhea, respectively. There were no severe adverse events observed in both groups during the study.

## Discussion

TCM has a well-documented history for treating infectious diseases during the past 3,000 years of Chinese history. In *Huangdi Neijing* (The Yellow Emperor's Classic of Medicine, an ancient treatise on health and disease) over 2,500 years ago, TCM was described to treat infectious diseases for the first time 15,16. COVID-19 was categorized as a cold and dampness epidemic 17, and the pathogenic factors were found in the body's mucous membranes 18. Dampness should be paid more attention to the epidemic 19. The complexity of COVID-19 lies in the fact that damp evil is dominant and dryness evil is contained 20. During the fight against COVID-19 in China, the National Health Commission of the People's Republic of China declared that 92% of the confirmed COVID-19 cases were treated with TCM in combination with the Western Medicine, and the patients responded to the treatment to recover or much improve in more than 90% of the cases 21. For patients with mild and moderate diseases, early intervention with TCM has been shown to effectively prevent disease transition into severe and critical state 22. TCM scheme has been included in the guideline on diagnosis and treatment of COVID-19 (Trial 9th edition) released by National Health Commission of the People's Republic of China 9.

JYGB has been prescribed for the treatment of upper respiratory tract illness, viral infection, and pneumonia 12,13. It has been recommended to treat patients with mild COVID-19 by the Expert consensus on TCM diagnosis and treatment of Coronavirus infection in Shanghai (2022 Spring Edition). To date, no previous studies have conducted to determine the efficacy of JYGB in patients with mild COVID-19. We found that when compared to control group, patients who received JYGB had a significant reduction in negative conversion time and a total length of hospital stay. The shortening of the negative conversion by one day is quite significant, especially when we dealt with the pandemic of this magnitude with limited resources such as the number of bed and healthcare personnel. This also allows a faster turnover of patients and enables us to admit new patients into quarantine and treatment. According to the previous study 23, COVID-19 patients with diabetes, chronic obstructive pulmonary disease, cardiovascular diseases, hypertension, malignancies, HIV, and other comorbidities could develop a life-threatening condition. In our study, the patients with comorbidity received the basic treatment of comorbidity during hospitalization, and there were no patients who developed into severe cases. We did not observe any differences in the proportion of patients with symptom improvement in both groups. In our sub-group analysis, JYGB is more effective in improving the negative conversion rate when it is administered ≤4 days from the onset of illness or in those ≤ 60 years old. We did not have a large number of patients who were > 60 years old, which limited our ability to determine the efficiency of the JYGB in these patients.

The mechanism of TCM in the treatment of COVID-19 is complex. COVID-19 can lead to a strong immune response and inflammatory storm 24. Administration of Chinese herbs may have beneficial immunomodulatory effects for rapid recovery of COVID-19 infections. JYGB takes banlangen, jinyinhua, and jingjie as the king medicine, huangqi, baizhu, and fangfeng as the minister medicine, huoixang, jiegeng, and lugen as the adjuvant, and gancao as the agent. Banlangen plays a role in directly killing pathogenic viruses or regulating the immune system to enhance anti-viral ability, which depends on the synergistic effects of its multiple components 25. Huangqi is used as immune stimulant, tonic, antioxidant, hepatoprotectant, diuretic, antidiabetic, anticancer, and expectorant 26. Astragaloside IV, the major component of Huangqi, is considered as an anti-inflammatory and antioxidant agent. Studies find that there is a significant overlap in GO terms and KEGG pathways between Astragaloside IV targets and SARS-CoV-2 DEGs, included MMP13, NLRP3, TRIM21, GBP1, ADORA2A, PTAFR, TNF, MLNR, IL1B, NFKBIA, ADRB2, and IL6, which suggests that Astragaloside IV maybe a new drug candidate for alleviating hyper-inflammation in COVID-19 patients 27. Platycodin D, a major component of Jiegeng, prevents both lysosome- and TMPRSS2-driven SARS-CoV-2 infection by hindering membrane fusion, which shows that is a potent natural product for preventing or treating COVID-19 28. More studies are needed to clarify the mechanisms of TCM.

One major limitation of this study is the mode of healthcare delivery for the treatment of patients with mild COVID-19, which is quite specific for the People's Republic of China. In other countries, patients with mild symptoms may not be hospitalized. We acknowledge the shortcomings to expand our results to other patient population.

In conclusion, we found that JYGB, a TCM prescription, improves the negative conversion rate and shortening the negative conversion time.

## Supplementary Material

Supplementary figures and tables.

## Figures and Tables

**Table 1:** Baseline characteristics of patients with mild COVID-19

Variables	Control Group (n=368)	Treatment Group (n=490)	*P value*
**Demographics**			
Male, n(%)	180 (48.91)	302 (61.63)	<0.001^***^
Age, Median (Q_1_, Q_3_), yr	48.0 (34.0, 57.0)	49.0 (34.0, 58.0)	0.30
n (%)			
<20	3 (0.82)	5 (1.02)	
20-	63 (17.12)	73 (14.90)	
30-	63 (17.12)	91 (18.57)	
40-	71 (19, 29)	78 (15.92)	
50-	105 (28.53)	142 (28.98)	
60-	49 (13.32)	79 (16.12)	
≥70	14 (3.80)	22 (4.49)	
BMI, Median (Q_1_,Q_3_), yr^A^	23.32(20.96, 25.39)	23.63 (21.45, 26.10)	0.06
n(%)			
<18.5	13 (3.70)	21 (4.60)	
18.5-23.9	191 (55.00)	221 (48.00)	
24.0-27.9	118 (34.00)	156 (33.90)	
>28	25 (7.20)	62 (13.50)	
**Characteristics**			
Symptoms, n (%)	311(84.51)	399 (81.43)	0.24
Fever	35 (9.51)	53 (10.82)	0.53
Cough	217 (58.97)	284 (57.96)	0.77
Hypodynamia	69 (18.75)	82 (16.73)	0.44
Headache	54 (14.67)	49 (10.00)	0.04^*^
Stuffy nose	84 (22.83)	126 (25.71)	0.33
Runny nose	75 (20.38)	94 (19.18)	0.66
Pharyngalgia	116 (31.52)	119 (24.28)	0.02^*^
Myalgia	28 (7.61)	32 (6.53)	0.54
Chest distress	27 (7.34)	28 (5.71)	0.34
Vomition	9 (2.45)	7 (1.43)	0.28
Abdominal distension	5 (1.36)	8 (1.63)	0.75
Stomachache	7 (1.90)	5 (1.02)	0.28
Diarrhoea	18 (4.89)	22 (4.49)	0.78
Comorbidity, n (%)	26 (7.07)	75 (15.31)	<0.001^***^
Hypertension	14 (3.80)	40 (8.16)	0.01^**^
Diabetes	9 (2.45)	17 (3.47)	0.39
Coronary artery heart disease	4 (1.09)	3 (0.61)	0.44
Chronic bronchitis	0 (0.00)	10 (2.04)	0.01^**^
Other diseases ^B^	6 (1.63 )	21 (4.29)	0.03^*^
Interval time^C^, Median(Q1,Q3), d	3.0 (2.0, 4.0)	3.0 (2.0, 4.0)	0.01^**^
Ct values of ORF gene^D^, Median (Q_1_, Q_3_)	25.26 (21.32, 29.76)	25.67 (22.15, 30.10)	0.29
Ct values of N gene^D^, Median (Q_1_, Q_3_)	24.49 (20.28, 28.81)	25.40 (21.74, 29.89)	0.004^**^

Note: A, Data were missing for 21 patients in the treatment group and 17 in the control group. BMI: body mass index. B, Other diseases included chronic liver diseases, chronic renal insufficiency, malignant tumor and rheumatic diseases. C,The interval time is the time between the onset of illness and randomization. Data were missing for 29 patients in the control group and 76 in the experiment group. The mean interval time in the control group is 2.83±1.65 and the mean interval time in the experiment group was 3.30±1.87 (*P*<0.001). D, The Ct values were gained in the first test of nucleic acid in mobile hospital.*, *P* ≤0.05; **, *P* ≤0.01; ***, *P*≤0.001.

**Table 2:** Cumulative negative conversion rates of SARS-CoV-2 RNA during treatment

During treatment	Control group (n=368)	Treatment group (n=490)	*P value*
Cumulative negative conversion rates, No./Total (%)			
At Day2	34/368 (9.24)	68/490 (13.88)	0.04^*^
At Day3	61/368 (16.58)	158/490 (32.24)	<0.001^***^
At Day4	133/368 (36.14)	252/490 (51.43)	<0.001^***^
At Day5	223/368 (60.60)	322/490 (65.71)	0.12
At Day6	257/368 (69.84)	381/490 (77.76)	0.008^**^
At Day7	304/368 (82.61)	427/490 (87.14)	0.06

Note: The levels of SARS-CoV-2 RNA were tested by real-time PCR every day after patients were treated. The cumulative negative conversion rates of SARS-CoV-2 were recorded every day. *, *P* ≤0.05; **P ≤0.01; ***P ≤0.001.

**Table 3:** The negative conversion time and the hospitalized days in all patients

Variables	Control group (n=368)	Treatment group (n=490)	*Z*	*P value*
Negative conversion time, Median (Q_1_, Q_3_), d	5.0 (4.0-7.0)	4.0 (3.0-6.0)	4.196	<0.001^***^
Hospitalized days, Median (Q_1_, Q_3_), d	7.0 (5.0-9.0)	6.0 (4.0-8.0)	4.884	<0.001^***^

Note: Negative conversion time and hospitalized days in all patients were analyzed. ***, *P* ≤ 0.001.

**Table 4:** Cumulative negative conversion rates of SARS-CoV-2 RNA in age subgroups during treatment

During treatment	Age ≤ 60 Age > 60					
	Control group (n=308)	Treatment group (n=397)	*P value*	Control group (n=60)	Treatment group (n=93)	*P value*
Cumulative negative conversion rate, No./Total (%)						
At Day2	30/308(9.74)	58/397 (14.61)	0.05^*^	4/60 (6.67)	10/93 (10.75)	0.39
At Day3	54/308(17.53)	136/397 (34.26)	<0.001^***^	7/60 (11.67)	22/93 (23.66)	0.07
At Day4	118/308(38.31)	209/397 (52.64)	<0.001^***^	15/60(25.00)	43/93 (46.24)	0.008^**^
At Day5	192/308(62.34)	275/397 (69.27)	0.05^*^	31/60(51.67)	47/93 (50.54)	0.89
At Day6	223/308(72.40)	319/397 (80.35)	0.01^**^	34/60(56.67)	62/93 (66.67)	0.21
At Day7	259/308(84.09)	352/397 (88.66)	0.08	45/60(75.00)	75/93 (80.65)	0.41

Note: Patients in every group were classified into age subgroups. The cumulative negative conversion rates were analyzed in the subgroup of age<60 and the subgroup of age≥60. *, *P* ≤0.05;**, *P* ≤0.01; ***, *P* ≤0.001.

**Table 5:** Negative conversion time and hospitalized days in age subgroups

Variables	Age ≤ 60				Age > 60			
	Control group (n=308)	Treatment group (n=397)	*Z*	*P value*	Control group (n=60)	Treatment group (n=93)	*Z*	*P value*
Negative conversion time, Median (Q_1_,Q_3_), d	5.0 (4.0, 7.0)	4.0 (3.0, 6.0)	-4.03	<0.001^***^	5.0 (4.3,7.8)	5.0 (4.0, 7.0)	-1.59	0.11
Hospitalized days, Median (Q_1_,Q_3_),d	7.0 (5.0, 9.0)	6.0 (4.0, 8.0)	-4.68	<0.001^***^	8.0 (6.0, 9.0)	7.0 (5.0, 9.0)	-1.67	0.09

Note: Negative conversion time and hospitalized days in age subgroups were analyzed. ***, *P*≤0.001.

**Table 6:** Cumulative negative conversion rates of SARS-CoV-2 RNA in the subgroups of interval time during treatment.

During treatment	Interval time ≤4 days			Interval time >4days		
	Control group (n=276)	Treatment group (n=320)	*P value*	Control group (n=63)	Treatment group (n=94)	*P value*
**Cumulative negative conversion rate, No./Total (%)**						
At Day2	20/276(7.25)	27/320(8.44)	0.59	13/63(20.63)	26/94(27.66)	0.32
At Day3	38/276(13.77)	75/320(23.44)	0.03^*^	22/63(34.92)	54/94(57.45)	0.006^**^
At Day4	88/276(31.88)	143/320(44.69)	0.01^**^	40/63(63.49)	70/94(74.47)	0.14
At Day5	163/276(59.06)	192/320(60.00)	0.82	50/63(79.37)	79/94(84.04)	0.45
At Day6	191/276(69.20)	238/320(74.38)	0.16	53/63(84.13)	84/94(89.36)	0.34
At Day7	229/276(82.97)	274/320(85.63)	0.37	57/63(90.48)	90/94(95.74)	0.20

Note: The interval time is the time between the onset of illness and randomization. Patients in every group were classified into subgroups according to interval time. The cumulative negative conversion rates were analyzed in the subgroup of interval time≤4 days and the subgroup of interval time>4 days. Among 858 patients, 753 patients who can provide the interval time from onset of illness to randomization were classified into subgroups according to interval time. *, *P* ≤0.05; **, *P* ≤0.01.

**Table 7:** Negative conversion time and hospitalized days in subgroups of interval time

Variables	Interval time ≤4 days				Interval time > 4days			
	Control group (n=276)	Treatment group (n=320)	*Z*	*P value*	Control group (n=63)	Treatment group (n=94)	*Z*	*P value*
Negative conversion time^A^, Median(Q_1_,Q_3_), d	5.0 (4.0,7.0)	5.0 (4.0,7.0)	2.35	0.02^*^	4.0 (3.0,5.0)	3.0 (2.0,5.0)	2.08	0.04^*^
Hospitalized days, Median(Q_1_,Q_3_), d	7.0 (6.0,9.0)	6.0 (5.0,9.0)	3.14	0.002^**^	6.0 (5.0,8.0)	5.0 (4.0,7.0)	2.41	0.02^*^

Note: Negative conversion time and hospitalized days in the subgroups of interval time were analyzed. *, *P* ≤0.05;^ **^, *P* ≤0.01. A, The average negative conversion time in the control group was 5.56±2.19 days, and that in the experiment group was 5.16±2.13 days, *P*=0.03.

## Abbreviation

Coronavirus disease 2019: COVID-19
JingYinGuBiao formula: JYGB
severe acute respiratory syndrome coronavirus 2: SARS-CoV-2
Traditional Chinese medicine: TCM
World Health Organization: WHO
