# Supplementary material for: Traditional Chinese Medicine JingYinGuBiao Formula Therapy Improves the Negative Conversion Rate of SARS-CoV2 in Patients with Mild COVID-19
Source: Int J Biol Sci. 2022 Sep 1;18(15):5641–52. doi: 10.7150/ijbs.76699 (PMC9576522; doi:10.7150/ijbs.76699)
Supplement: Supplementary file 1 — Supplementary figures and tables. [file ijbsv18p5641s1.pdf]

Supplemental data

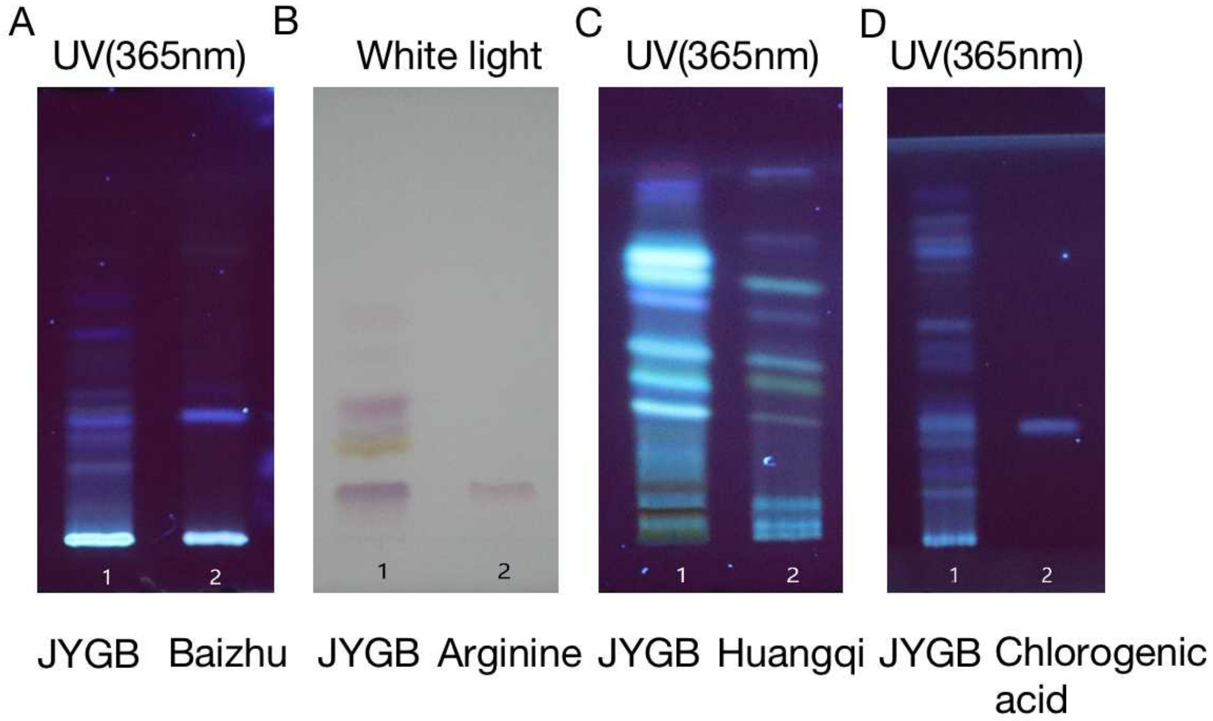

Figure S1 The quality control of herbs

According to the Chinese Pharmacopoeia (2020 Edition), samples were tested by the thin-layer identification method. Both TCM granules and the control samples can show the spots of the same color at RF value. A. The results of JYGB granules and its control sample(Baizhu) were shown. B. The results of JYGB granuels and its control sample(Arginine) were shown. C. The results of JYGB granules and its control sample(Huangqi) were shown. D.The results of JYGB granules and its control smaple(Chlorogenic acid) were shown.

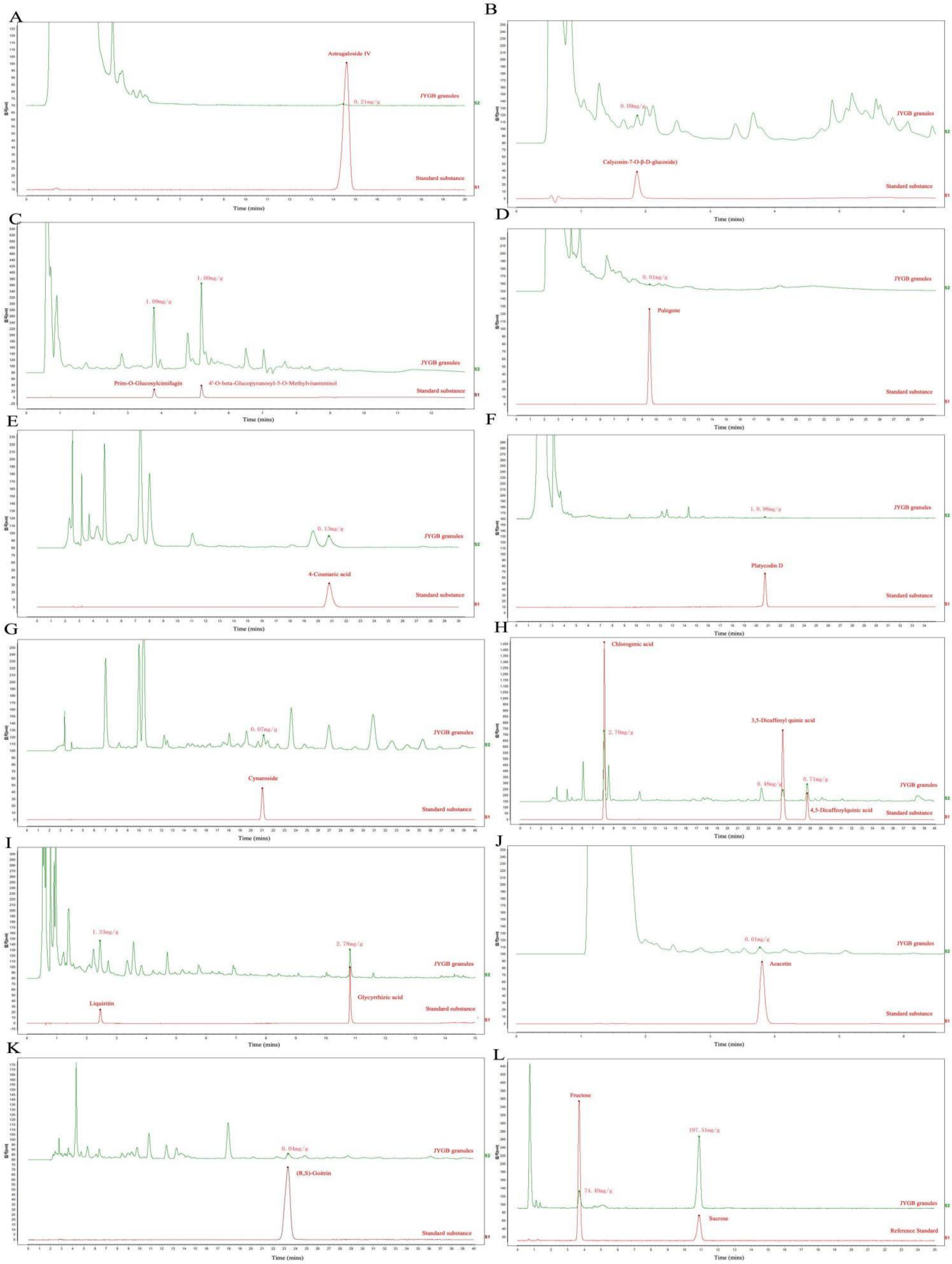

Figure S2 The HPLC-MS ionic chromatographic fingerprints of the components in JYGB granules

A-F, The components in JYGB granules were identified by HPLC-MS, and the HPLC-MS ionic chromatographic fingerprints of the components in JYGB granules were shown.

Table S1 Chinese Simplified Script and English Translations of Traditional Chinese in this article*

English Translation	Chinese Simplified script	Traditional script	English	Latin
Jingyingubiao formula	荆银固表方	荆银固表方	/	/
King medicine	君	君	/	/
Minster medicine	臣	臣	/	/
Assistant medicine	佐	佐	/	/
Ambassador medicine	使	使	/	/
Jinyinhua	金银花	金銀花	Los Lonicera	Lonicera japonica Thunb
Jingjie	荆芥	荊芥	Schizonepeta	Schizonepeta species
Huangqi	黄芪	黃芪	Milkvetch Root	Astragalus membranaceus
Fangfeng	防风	防風	Divaricate Saposhniovia Root	Radix Saposhnikoviae
Huoxiang	藿香	藿香	Cablin Potchouli Herb	Agastache rugosa
Banlangen	板蓝根	闆藍根	Indigowoad Root	Isatis tinctoria
Jiegeng	桔梗	桔梗	Balloonflower	Platycodon gradiflorus
Lugen	芦根	蘆根	ReedRhizome	Rhizoma Phragmitis
Baishu	白术	白術	Largehead Atractylodes Rh	Atractylodes macrocephala
Gancao	甘草	甘草	liquorice root	Glycyrrhiza uralensis Fisch

Note: * Chinese herbs are usually prescribed in formulas that contain "king" medicines, which provide the strongest therapeutic action; "minister" medicines, which assist the "king" medicine in its actions; "assistant" medicines, which aid the"minister" medicine in treating a lesser aspect of the disease; and "ambassador"medicines that are intended to reduce the toxicity of the other medicines in the formula or guide the formula to the targeted organ or region of the body[1].

1. Cheng JT. Review: drug therapy in Chinese traditional medicine. J Clin Pharmacol. 2000;40:445-50. [PMID: 10806595]

Table S2 The identification analysis of the components in JYGB granules

Herb name	Compound name	Molecular formula	Molecular structure	Content in JYGB granules
Huangqi (Astragalus propinquus Schischkin)	Astragaloside IV	$C_{41}H_{68}O_{14}$	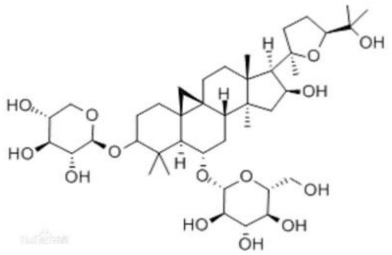	0.21mg/g
Huangqi(Astragalus propinquus Schischkin)	Calycosin-7-glucoside	$C_{22}H_{22}O_{10}$	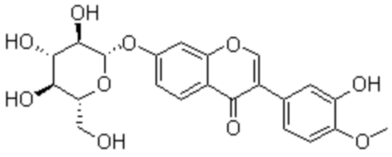	0.09mg/g
Fangfeng(Saposhnikovia divaricate)	Prim-O-glucosylcimifugin	$C_{22}H_{28}O_{11}$	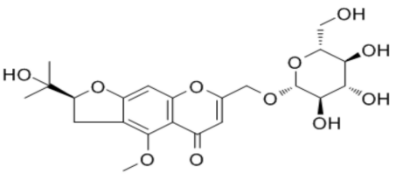	1.09mg/g
Fangfeng(Saposhnikovia divaricate)	4'-O-beta-Glucopyranosyl-5-O-Methylvisamminol	C22H28O10	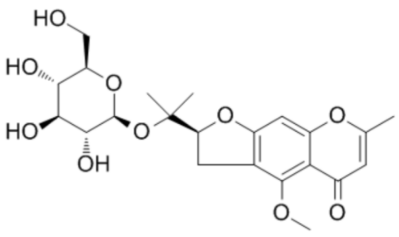	1.00mg/g
Jingjie (Herba Schizonepetae)	Pulegone	C10H16O	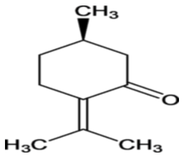	0.01mg/g
lugen (Rhizoma phragmitis)	4-Coumaric acid	C9H8O3	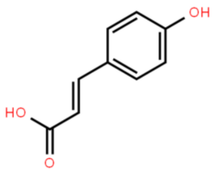	0.13mg/g
Jiegeng (Platycodon Grandiflorum),	Platycodin D	C57H92O28	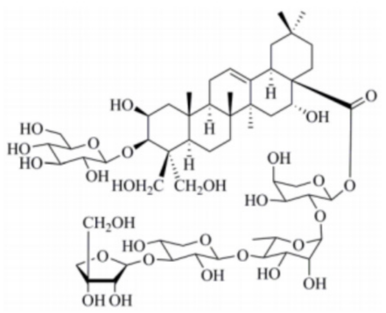	0.98mg/g

Jinyinhua (Lonicera japonica Thunb)	Cynaroside	C21H20O11	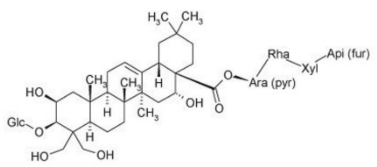	0.07mg/g
Jinyinhua (Lonicera japonica Thunb)	Chlorogenic acid	C16H18O9	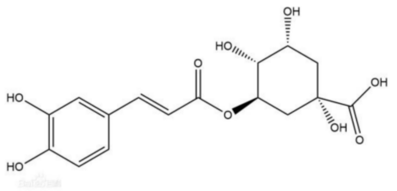	2.70mg/g
Jinyinhua (Lonicera japonica Thunb)	3,5-O dicaffeo ylquinic acid	C25H24O12	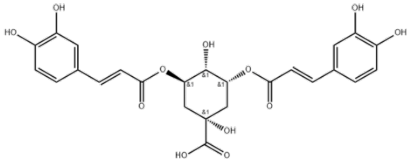	0.48mg/g
Jinyinhua (Lonicera japonica Thunb	4,5-Dicaffeoylq uinic acid	C25H24O12	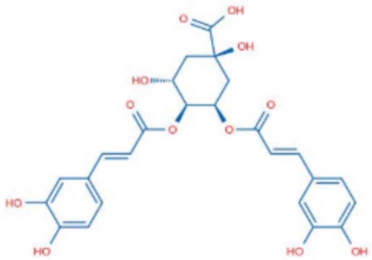	0.71mg/g
gancao (Glycyrrhizaural ensisFisch)	Liquiritin	C21H22O9	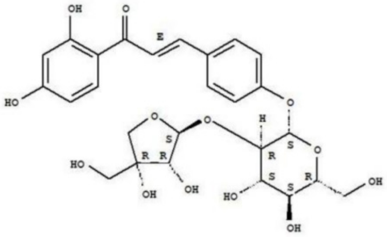	1.33mg/g
gancao (Glycyrrhizaural ensisFisch)	Glycyrrhizic acid	C42H62O16	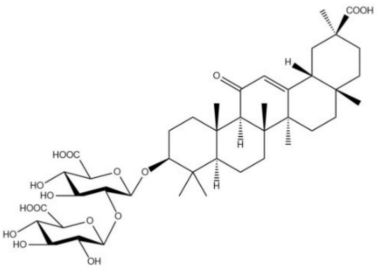	2.78mg/g
huoxiang (Agastache rugosus)	Acacetin	C16H12O5	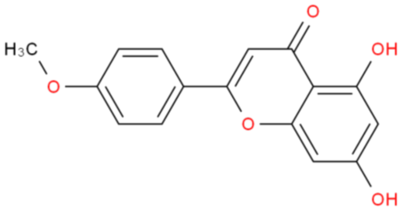	0.01mg/g
banlangen (Isatis Root)	(R,S)-Goitrin	C5H7NOS	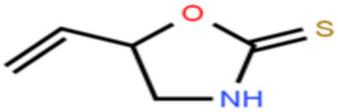	0.04mg/g

baishu (Atractylodes macrocephala Koidz)	fructose	C6H12O6	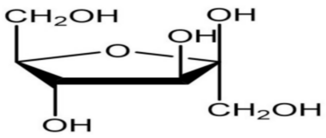	74.40mg/g
baishu (Atractylodes macrocephala Koidz)	Sucrose	C12H22O11	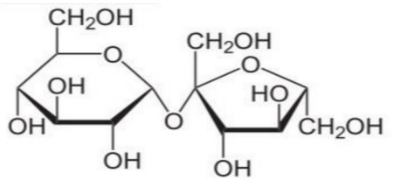	197.51mg/g

Note. An ultra-high performance liquid chromatography-quadrupole/Orbitrap high resolution mass spectrometry (UHPLC-Q-Orbitrap HRMS) was used for the identification analysis of the components in JYGB granules, and the multistage fragments ions data was compared with the standard substance and literature consulting.

Table S3 The improvement rate of symptoms after treatment

Symptoms	Treatment group No./Total (%)	Control group No./Total (%)	Total No./Total(%)	*P* value
Fever	51/53(96.23)	33/35(94.29)	84/88(95.45)	1.00
Cough	221/284 (77.82)	154/217(70.97)	375/501(74.85)	0.08
Hypodynamia	75/82 (91.46)	59/69(85.51)	134/151(88.74)	0.25
Headache	46/49 (93.88)	52/54(96.30)	98/103(95.15)	0.91
Stuffy nose	109/126 (86.51)	79/84(94.05)	188/210(89.52)	0.08
Runny nose	88/94(93.62)	71/75(94.67)	159/169(94.08)	0.77
Pharyngalgia	105/119(88.24)	105/116(90.52)	210/235(89.36)	0.57
Myalgia	31/32(96.88)	26/28(92.86)	57/60(95.00)	0.91
Chest distress	26/28(92.86)	26/27 (96.03)	52/55 (94.55)	0.58
Vomition	7/7(100.00)	9/9(100.00)	16/16(100.00)	NA
Abdominal distension	8/8(100.00)	5/5(100.00)	13/13(100.00)	NA
Stomachache	5/5(100.00)	7/7(100.00)	12/12(100.00)	NA
Diarrhoea	21/22(95.45)	15/18(83.33)	36/40(90.00)	0.46

Table S4 Univariate and multivariate Cox regression model for negative conversion of SARS-CoV2

	Univariate analysis			Multi-variate analysis		
**Variables**	**ORs**	**95%CI**	***P* value**	**ORs**	**95%CI**	***P* value**
Treatment (JYGB)	1.23	1.07-1.40	0.003	1.18	1.02-1.37	0.03
Gender	0.98	0.86-1.12	0.76			
Age	0.99	0.99-1.00	0.008			
Age Group	0.95	0.90-0.99	0.02	0.92	0.87-0.97	0.001
BMI	1.00	0.98-1.02	0.99			
BMI Group	1.03	0.94-1.13	0.55			
Symptoms (Yes)	0.81	0.68-0.97	0.02			
Combidities(Yes)	1.06	0.86-1.30	0.59			
Interval time	1.19	1.14-1.24	<0.001	1.18	1.11-1.27	<0.001
Ct of *ORF1* gene	1.09	1.08-1.11	<0.001	1.07	1.06-1.09	<0.001
Ct of *N* gene	1.09	1.07-1.10	<0.001			
